# Adolescents’ Experiences of Using a Smartphone Application Hosting a Closed-loop Algorithm to Manage Type 1 Diabetes in Everyday Life: Qualitative Study

**DOI:** 10.1177/1932296821994201

**Published:** 2021-07-14

**Authors:** David Rankin, Barbara Kimbell, Janet M. Allen, Rachel E. J. Besser, Charlotte K. Boughton, Fiona Campbell, Daniela Elleri, Julia Fuchs, Atrayee Ghatak, Tabitha Randell, Ajay Thankamony, Nicola Trevelyan, Malgorzata E. Wilinska, Roman Hovorka, Julia Lawton

**Affiliations:** 1 Usher Institute, Medical School, University of Edinburgh, UK; 2 Wellcome Trust – Medical Research Institute of Metabolic Science, University of Cambridge, UK; 3 Department of Paediatrics, University of Cambridge, UK; 4 NIHR Oxford Biomedical Research Centre, Oxford University Hospitals NHS Foundation Trust, UK; 5 Department of Paediatrics, University of Oxford, UK; 6 St James’s University Hospital, Leeds, UK; 7 Royal Hospital for Sick Children, Edinburgh, UK; 8 Alder Hey Children’s NHS Foundation Trust, Liverpool, UK; 9 Nottingham Children’s Hospital, UK; 10 Addenbrookes Hospital, Cambridge University Hospitals NHS Foundation Trust, UK; 11 Southampton Children’s Hospital, UK

**Keywords:** closed-loop, continuous glucose monitor, disparities, patient experiences, qualitative methods, type 1 diabetes

## Abstract

**Background:**

Closed-loop technology may help address health disparities experienced by adolescents, who are more likely to have suboptimal glycemic control than other age groups and, because of their age, find diabetes self-management particularly challenging. The CamAPS FX closed-loop has sought to address accessibility and usability issues reported by users of previous prototype systems. It comprises small components and a smartphone app used to: announce meal-time boluses, adjust (“boost” or “ease-off”) closed-loop insulin delivery, customize alarms, and review/share data. We explored how using the CamAPS FX platform influences adolescents’ self-management practices and everyday lives.

**Methods:**

Eighteen adolescents were interviewed after having ≥6 months experience using the closed-loop platform. Data were analyzed thematically.

**Results:**

Participants reported feeling less burdened and shackled by diabetes because closed-loop components were easier to carry/wear, finger-pricks were not required, the smartphone app provided a discreet and less stigmatizing way of managing diabetes in public, and they were able to customize alarms. Participants also reported checking and reviewing data more regularly, because they did so when using the smartphone for other reasons. Some reported challenges in school settings where use of personal phones was restricted. Participants highlighted how self-management practices were improved because they could easily review glucose data and adjust closed-loop insulin delivery using the “boost” and “ease-off” functions. Some described how using the system resulted in them forgetting about diabetes and neglecting certain tasks.

**Conclusions:**

A closed-loop system with small components and control algorithm on a smartphone app can enhance usability and acceptability for adolescents and may help address the health-related disparities experienced by this age group. However, challenges can arise from using a medical app on a device which doubles as a smartphone.

**Trial registration:**

Closed Loop From Onset in Type 1 Diabetes (CLOuD); NCT02871089; https://clinicaltrials.gov/ct2/show/NCT02871089

## Introduction

A closed-loop system is a rapidly evolving technology for people with diabetes, which requires varying degrees of user input; for example, to calibrate the continuous glucose monitor (CGM) or count carbohydrates and input this information prior to eating. Trials of early iterations of this technology were undertaken overnight, with the control algorithm hosted on a laptop or tablet.^1,2^ More recently, trials in real-life settings have investigated day-and-night use with the algorithm hosted on the pump itself, or on a portable handheld device or smartphone,^3,4^ including the Medtronic 670G^5^ and the Tandem T-slim pump with Control-IQ,^6^ which are used in clinical practice in the United States and Europe.

Health-related disparities are often attributed to demographic factors, such as socioeconomic status or ethnicity, but can also be age related.^7^ Adolescents with type 1 diabetes experience higher HbA1c levels than individuals in other age groups,^8^ leading to an increased risk of developing diabetic ketoacidosis^9^ and premature mortality.^10^ These age-related health disparities have been attributed to factors that make diabetes self-management particularly challenging in this age group, including peer group influences, family conflict, greater risk-taking, and “diabetes burnout.”^11-14^ Adolescents also experience greater prevalence of anxiety, depression, and disordered eating,^15^ which can further compound difficulties undertaking self-management tasks and ensuing health disparities.

Evaluations of closed-loop technology involving adolescents have demonstrated improved blood glucose control and decreased frequency of hypoglycaemia,^1,2,4,16-21^ including in adolescents with suboptimal glycemic control.^18^ While this kind of technology therefore has potential to address health-related disparities in the adolescent age group, barriers to using this and related (eg, CGM, pump) technology have been reported, which can lead to discontinuation or low closed-loop use among adolescent users.^20,22-25^ These include concerns about: wearing large CGM and/or pump components^25-27^; using bulky phone handsets that are awkward to carry and easy to forget, often resulting in system connectivity issues^21-23^; needing to perform excessive finger-prick checks, including to calibrate the CGM^20,25-29^; and “alarm fatigue” resulting from these being too frequent and intrusive.^20,25-30^ Adolescents, like adult users,^31^ have also expressed a desire to collaborate with the system by being able to instruct the algorithm when routines change or an atypical day is planned.^13,29,31^

A recently developed iteration of closed-loop technology, CamAPS FX,^32^ has taken account of user feedback and comprises: a CGM (Dexcom G6) that does not require calibration, a smaller pump (Dana RS), and a control algorithm hosted within an app on an unlocked Android smartphone. The closed-loop app includes functions that enable users to: announce meal-time boluses, issue commands to increase (“boost”) or decrease (“ease-off”) closed-loop insulin delivery, switch on/off and customize alarms, review their data history onscreen, and share data in “real-time” with health professionals and parents/caregivers. In this article, we report findings from a qualitative evaluation involving adolescents who had ≥6 months experience of using the CamAPS FX platform. Specifically, we sought to explore how using the closed-loop system influences adolescents’ self-management practices and lives.

## Methods

We used an inductive, semi-structured interview design that enabled the discussion to remain relevant to addressing the study aims while giving participants flexibility to discuss issues they considered salient, including those unforeseen at study outset. A topic guide was used (see Table 1), which was informed by literature reviews and inputs from patient representatives and clinical co-investigators. Data collection and analysis took place concurrently so that findings identified in early interviews could inform the topics explored in later accounts.

**Table 1.** Relevant Areas Explored in Interviews.

• Background information: age, living arrangements, everyday school and family life; interests and hobbies, views about using technology in everyday life.
• Views about and experiences of using the CamAPS FX closed-loop to manage diabetes in everyday life at and away from home:
- Inserting cannulas and CGM sensors, using the study pump, wearing devices on the body, using the study smartphone and app; ensuring devices are connected.
- Using the app to monitor blood glucose levels; using finger-pricks to monitor blood glucose.
- Using data on app to inform decisions about diabetes management tasks (eg, insulin administration, treating hypoglycemia, responding to high glucose levels).
- Managing hypo- or hyperglycemia.
- Reasons for using the “Boost” and “Ease-off” functions; use of corrective doses of insulin.
- Reasons for activating/deactivating and/or customizing system-generated alarms; and responses to these.
• Perceived impact of using the closed-loop on self-perceptions, relationships with others and everyday life, including when at school or in other educational settings.

Abbreviation: CGM, continuous glucose monitor.

### Recruitment and Data Collection

Individuals were recruited following randomization to a 24-month open-label, UK-based, multicenter trial, followed by an optional 24-month extension phase, designed to assess the clinical effects of closed-loop insulin delivery compared with a multiple daily injection (MDI) regimen, in youths (aged 10-16.9 years) from onset of type 1 diabetes.^32^ Participants in the intervention arm began by using the previous prototype, FlorenceM closed-loop, before transiting to the CamAPS FX platform; both systems used the same control algorithm (see Table 2 for more detail about the trial and closed-loop systems).

**Table 2.** Trial Eligibility Criteria and Description of Closed-loop Systems.

*Trial eligibility criteria*	
Eligibility criteria for the trial included: a diagnosis of type 1 diabetes within the preceding 21 days; aged between 10 and 16.9 years old; and a willingness to perform capillary blood glucose monitoring (at least four blood glucose measurements each day), wear study devices (glucose sensor and closed-loop system), and upload pump and CGM data at regular intervals.	
*Closed-loop systems used during the trial*	
Participants in this qualitative study began by using the FlorenceM hybrid closed-loop system before transiting to the CamAPS FX hybrid closed-loop platform. The control algorithm is the same for both systems.	
*FlorenceM hybrid closed-loop system*	*CamAPS FX hybrid closed-loop platform*
• Modified next generation sensor augmented Medtronic insulin pump 640G (Medtronic, Northridge, CA, USA) with CGM receiver and low glucose suspend feature.	• Dana RS insulin pump (Diabecare, Sooil, Seoul, South Korea).
• Guardian 3 sensor (Medtronic).	• Dexcom G6 real-time CGM sensor (Dexcom, San Diego, CA, USA).
• A *locked-down* Android smartphone (Galaxy S4, Samsung, South Korea) hosting the Cambridge model predictive control algorithm (University of Cambridge, Cambridge, UK) connected to a propriety translator to allow wireless communication with the insulin pump.	• An *unlocked* Android smartphone (Galaxy S8, Samsung, South Korea) running Android 5.0 OS or above, which hosted the CamAPS FX Application incorporating the Cambridge model predictive control algorithm (University of Cambridge, Cambridge, UK) and communicating wirelessly with the insulin pump. Participants could opt to use their personal smartphone if compatible.
• FlorenceM Users were required to: use a standard bolus calculator on the pump to enter information about carbohydrates and deliver meal boluses; change their pump infusion set every 2-3 days; replace the CGM sensor at least every 7 days; perform four finger-pricks per day before meals and to calibrate the sensor as required; respond to alarms alerting them to high/low blood glucose or if they needed to recalibrate the sensor; ensure that study devices (CGM transmitter and smartphone) are charged; and ensure the smartphone is kept in close proximity (5-10 meters) to avoid signal loss with the pump/CGM. Users could choose to activate an “exercise” function on the pump to deliver an extended bolus dose of insulin during periods of physical activity.	• CamAPS FX Users were required to: use the bolus calculator on the app to deliver meal boluses; change their pump infusion set every 2-3 days; replace the CGM sensor at least every 10 days; respond to alarms alerting them to high/low blood glucose; ensure that study devices (smartphone) are charged; and ensure the smartphone is kept in close proximity (5-10 m) to avoid signal loss with the pump/CGM. Further detail about the CamAPS FX app is provided below.
*CamAPS FX app*	
In addition to being used to administer mealtime boluses of insulin, the app included functions enabling users to:	
• view a “real-time” graph displaying their sensor glucose levels, rate of insulin delivery, meal-time boluses and carbohydrate intake, high/low glucose range, glucose trend arrows, whether “boost” or “ease-off” functions were activated (see below), and whether the closed-loop was operational (Auto mode on) or interrupted (Auto mode off).	
• view summary statistics for daily, weekly, monthly, or three-monthly periods, including: average glucose, estimated HbA1c, time in/below/above target, number and average duration of hypos, total daily dose/bolus/basal insulin, and percentage of time in Auto Mode.	
• issue instructions to the closed-loop to initiate a “Boost” mode of operation when more insulin was needed (eg, during periods of inactivity, increased food intake, or during illness or stress), or an “Ease-off” mode when less insulin was needed (eg, during exercise or when glucose levels tend to be low).	
• receive and personalize alarms/alerts triggered by high/low glucose, and signal loss with the sensor and/or pump, by adjusting the threshold, repeat time and audio sound or vibration, which accompanied an on-screen display, and turn on/off all alerts (except the “Urgent Low” glucose alarm).	
• share (by automatically uploading to the cloud) with health professionals, and their parents/caregivers, near “real-time” glucose levels, insulin, and meal-time bolus data, which could be accessed using the Diasend/Glooko app, and an option to relay alarms activated by users to a user-determined set of “followers” alerting them to high/low blood glucose, sent via SMS texts.	

Abbreviation: CGM, continuous glucose monitor.

Participants randomized to the closed-loop arm were recruited into the interview study by health professionals from six participating UK sites using an opt-in procedure. Purposive sampling was used to ensure diversity in adolescents’ gender, age, and parental occupation (as a proxy for socioeconomic status). Recruitment and data collection continued until no new findings were identified in new data collected (data saturation). Interviews were conducted by DR, an experienced nonclinical qualitative researcher. DR made it clear to participants that he was an independent researcher and that their participation in the interview study would not affect their clinical care. This information was provided to help ensure participants felt able to share negative views about using the closed-loop should they wish to do so. Interviews were conducted at a time of participants’ choosing between November 2019 and March 2020. These were audio-recorded, lasted 1-1.5 h, and were transcribed in full.

The study received approval from Cambridge East Research Ethics Committee (REC ref: 16/EE/0286) and the Medicines & Health products Regulatory Agency.

### Data Analysis

The interviews were analyzed by DR and JL using the method of constant comparison^33^ to identify cross-cutting themes. Both researchers read interviews repeatedly before undertaking preliminary analyses and writing separate reports. Regular meetings were held to compare interpretations and reach agreement on a coding framework, which captured key themes. A qualitative data-indexing package, Nvivo11 (QSR International, Doncaster, Australia), was used to facilitate data coding and retrieval. Coded datasets were subjected to further analyses to allow more nuanced interpretations of the data and identify illustrative quotations.

## Results

The sample comprised 18 adolescents, and demographic data are presented in Table 3. We identified several cross-cutting themes that illustrate how using the closed-loop platform: lessened the burdens of self-management and helped to normalize participants’ lives; facilitated opportunities to monitor and review data; enabled them to collaborate with the system to optimize glucose control; and provided options to customize alarms. We also report participants’ accounts of unintended consequences resulting from using the system. When reflecting on their experiences of using the CamAPS FX platform participants drew on earlier experiences using the FlorenceM closed-loop, which was their main basis for comparison following diagnosis and recruitment to the trial.

**Table 3.** Demographic Characteristics of Study Participants.

Characteristic	*N*	%	Mean ± SD and range
*Adolescents (n = 18)*			
Female	8	44.4	
Age at interview—all children (years)			14.3 ± 2.1, range 11-18
Diabetes duration (months)			23.7 ± 6.6, range 16-34
Duration using FlorenceM (months)			16.6 ± 6.5, range 8-27
Duration using CamAPS FX (months)			6.3 ± 0.5, range 6-7
*Ethnicity*			
White, British	18	100	
*Parents (n = 32)*			
Occupation			
Professional	15	46.9	
Semi-skilled	13	40.6	
Unemployed/full-time carer	4	12.5	

Abbreviation: SD, standard deviation.

### Benefits: Less Work, Feeling More Normal, and Less Stigmatized

All participants reported needing to do “much less work” (Participant#3) using the new closed-loop system because the G6 sensor gave “really accurate” (Participant#9) glucose readings, which meant they did not need to perform finger-pricks for calibrations, before meals, after correcting high readings or treating hypoglycemia. As they highlighted, using the app to check glucose levels resulted in them feeling less shackled by diabetes because “you don’t have to be taking round bits of stuff, like a testing kit” (Participant#2) and “I don’t do any [finger-pricks] at all which is better and then they [parents] don’t have to nag me to do them” (Participant#6). Individuals also described how relationships with parents had improved because they could remote monitor their data and, hence, no longer burdened them with requests to provide this information (see Table 4).

**Table 4.** Participant Quotations.

Theme	Participant quotations
Benefits: less work, feeling more normal, and less stigmatized	*Benefits of parents being able to remotely monitor data*
	it was quite annoying, because it [data] was only on my phone [when using FlorenceM]. So if they wanted to look at it, I’d have to stay there for a bit and obviously be next to the phone while they’re looking at it. But it’s just easier on theirs [phone], so that they’re not always asking about, you know, ‘let me see your phone, let me just check this for a minute’. It’s just easier on their phone (Participant#5)
	*Less work and effort*
	you can do like boluses, like on the go. . . . So like say you’re walking in town or something, you can just look through it [closed-loop app] there. . . instead of having to like sit down and take it [pump] out. I think it’s a lot more like suited to maybe modern lifestyle (Participant#2)
	*More discreet, less stigmatizing*
	I used to have to you know, take my pump out, like you could obviously see it, whereas now it only looks like I’m on my phone, you know, playing a game or something. . . it doesn’t make as big a deal of it, you know, if I’m out [in public] or something (Participant#5).
Better opportunities to monitor and review glucose readings	*Monitoring and reviewing glucose levels*
	I just take it [phone] everywhere with me. . . So when it’s [closed-loop app] in my phone, I have that with me anyway, all the time. And I’m mostly on it all the time, like talking to my mum, or talking to my friends. So it’s easy to do it [check glucose levels] (Participant#9).
	*Reviewing data to inform adjustments to insulin doses*
	I have a look back at me day, see if I’ve been high or low. . . if I’m in school, I’ll have a look at the lunchtime and the break-time if I eat. And then if it’s been high the day before, I’d make sure I put in a bit of extra carbs the next day (Participant#12).
	*Use of closed-loop in schools*
	sometimes in class I have checked but I’ve not like tooken [taken] my phone out of my bag, I’ve just looked, like I’ve just did it like, had the phone in my bag and just like done it through putting my hand in my bag and like act like I’m looking for something or something like that (Participant#10)
Better glycemic control: collaborating with the closed-loop using Boost and Ease-off	*Boost: better than using corrections*
	I kept having to like, I’d get my pump out [when using the previous closed-loop], do a correction, wait a few, like 20, 30 minutes. Then I’d check it [glucose level on pump] and I’d be like, ‘oh, I still haven’t gone down yet’, so I’d have to do corrections, corrections, corrections. Whereas with this one [CamAPS FX], I put a Boost on. . . I’d check my phone and then I just see if I’ve come down (Participant#9)
	*Boost: dealing with post-prandial high blood glucose levels*
	if I know what I’m eating, like a high-carb meal and I’m gonna go high after it no matter what I take, I can put ‘Boost’ on for it. . . like pasta, chips, em bread. . . With the ‘Boost’ on, it’ll increase the temp basal, which is very helpful (Participant#13)
	*Ease-off: less concerns about developing hypoglycemia*
	if I was doing PE at school [using the previous closed-loop], then I kept having to worry about going low and things like that, which sometimes happen if I’m doing quite intensive sports. But if I was to have eased-off [before doing physical activity] then I wouldn’t worry about them [hypos] so much (Participant#18)
Alarms	*Customizing alerts: less stigma*
	In school I keep my phone [alarms] on vibrate. . . only I can hear it because it’s on my chair. . . So it’s a lot less of a you know, it like it doesn’t make a scene or anything . . . it makes it better because nobody actually knows (Participant#7)
	*Customizing and responding to alarms*
	I’ve turned off the alarms for going high. . . it kept going off when I felt I had it under control, when I didn’t feel like I needed to be reminded. It sort of meant I’d hear an alarm and not really react. . . Whereas, because I turned it off, it’s like if I hear an alarm now, I know it’s because I’m considerably low, so it’s something I really need to check out. (Participant#16)
Unintended consequences: over-reliance on the closed-loop	*Less time thinking about diabetes*
	it improves the way that your life with diabetes is, because you’ve got such good equipment to control it, there’s not really much things like finger-pricks or injections to do. . . also because I’ve got a normal phone, and it’s my day-to-day phone. And the pump’s a lot lighter, so hardly thinking about that. And my sensor’s so easy to put on. So just things like that decrease the amount you think about it [diabetes] (Participant#17)
	*Forgetting to perform key tasks*
	it’s almost too easy. It’s – it’s a victim of like its own success in that way, in that like it’s so easy that maybe you don’t pay full attention. . . or you’re not hyper like concentrated and worried. Like I think that’s a good thing in some ways but it’s sort of, like, it’s too good, so maybe your focus isn’t quite there. (Participant#16)
	Interviewer: “In what ways do you feel that your focus might be affected by the system being as good as it is?”
	yeah, yeah someti- I will sometimes em, I might sometimes just forget to put in the bolus, but it’s not- I don’t do it particularly often, but I do sometimes- sometimes if I’m tired I will. (Participant#16)

Most also observed how diabetes had receded into the background by virtue of the G6 sensor being easier to wear: “it stays on so much better, it’s just smaller” (Participant#3) and only needing to be replaced every 10 days, while the Dana RS pump was “smaller and lighter” (Participant#16), “easier to fit in your pocket” (Participant#4), and did not need to be accessed to administer insulin. Indeed, many recalled the cumbersome process of having to “untangle yourself” (Participant#1) or “fumble about in your pocket” (Participant#3) to retrieve their pump to manage diabetes using the previous system, whereas the app enabled them to check glucose levels and administer insulin effortlessly (see Table 4). Participants also noted how having the closed-loop app reside on a smartphone offered a more discreet and less stigmatizing way of performing these tasks in public, because other people construed that they were undertaking normal activities, such as checking the time or texting (see Table 4).

### Better Opportunities to Monitor and Review Glucose Readings

All participants noted how having the app on an unlocked smartphone, which they could use as their personal phone, meant it was always kept close-to-hand or carried on their person. As a result, they described using the app to monitor their glucose levels more frequently than had happened when using the previous system because this could be done opportunistically while using the phone for other reasons, such as checking social media or making calls (see Table 4). Participants also reported how having the app on a smartphone meant they had easy access to a graph, which displayed a real-time record of their glucose levels, insulin on board, and carbohydrate intake: “if you need to take your insulin, or have a look at your bloods, you can see your chart [graph]” (Participant#13). As they noted, reviews of these data helped inform adjustments, such as identifying times to take preventative quick-acting carbohydrate to counter falling glucose levels, or refining carbohydrate calculations for meals (see Table 4). Others observed that always carrying the study phone meant they did not experience the connectivity issues encountered using the previous system, as components were more likely to be in range, connected, and operating in Auto mode.

While most participants reported similar benefits when using the app to manage diabetes in schools, several indicated that mobile phone policies had created difficulties, particularly if teachers were not familiar with reasons for needing to consult the app during lessons. As a result, some described using the app covertly: “I just find it easier just doing it under the table” (Participant#18), or checking their glucose levels less often during classes because they were not permitted to have a phone on their desk (see Table 4).

### Better Glycemic Control: Collaborating with the Closed-loop Using “Boost” and “Ease-off”

When reflecting on their earlier experiences, participants described being able to better manage diabetes and optimize glycemic control with the CamAPS FX system because they could use the “boost” and “ease-off” functions.

#### Boost

Individuals noted that the “boost” function provided them with “a lot more options to deal with highs” (Participant#7), including elevated post-prandial glucose levels, compared with their experiences using the previous system and having to calculate, administer, and check the outcome after inputting corrective doses of insulin (see Table 4). Participants also described being able to better manage glucose excursions resulting from eating high-carbohydrate foods. This was because they could “boost” if their glucose levels had risen too high or use the function in advance of eating high-carbohydrate foods to assist the algorithm’s response to rising glucose levels (see Table 4).

Others reported that using “boost” helped them achieve better glycemic control if they felt unwell or if their glucose levels were unexpectedly high and the algorithm had yet to bring them into range: “if I’m sick one day or I’m running high for no reason, I’ll put a ‘Boost’ on. . . I’ve noticed that [when I’m high] if I don’t have a Boost on, I probably would go higher” (Participant#17)

#### Ease-off

Very few participants recalled using the temporary basal “exercise” setting on the pump component forming part of the previous system; however, most described routinely using the “ease-off” function on the closed-loop app prior to physical activity because: “it’s [‘ease-off’] just a lot simpler and easier to use . . . all of the configuration or anything you need to do is on the app itself” (Participant#3). As individuals noted, being able to use this function resulted in them feeling less worried about developing hypoglycemia when they were physically active (see Table 4).

### Alarms

All participants highlighted the benefits of no longer receiving calibration alerts, which helped lessen disruptions at night: “this system gives you alarms only if you really need them, so this one’s definitely better for like sleep” (Participant#10). As others reported, being able to easily silence alarms helped limit the risk of being stigmatized by peers at school (see Table 4).

Many also described benefits from being able to customize the glucose levels, which would trigger alarms: “I set an alert if my blood sugar like say is 4.8, it’ll give me a notification, so I can drink some Lucozade. . . before it actually goes like really low” (Participant#3) or by deactivating high glucose alerts if they felt confident the closed-loop would bring their levels into range. Indeed, as several pointed out, being able to configure alarms made it more likely that they would respond appropriately when those they had activated were triggered (see Table 4).

### Unintended Consequences: Over-reliance on the Closed-loop

While participants noted that the closed-loop platform had alleviated much of the effort and burden associated with diabetes management, several indicated that this meant they spent less time thinking about diabetes in general (see Table 4). These individuals also described how this had resulted in them sometimes forgetting to carry out key tasks such as administering a bolus for a meal (see Table 4).

## Discussion

The CamAPS FX system^32^ addresses many of the barriers to access and usability issues reported by (adolescent) users of previous closed-loop systems,^20,25-29^ CGMs,^24,34^ and insulin pumps.^35,36^ Usability is a key factor in determining closed-loop use, and higher usage has been shown to be associated with improved glycemic outcomes and lower HbA1c.^20,21^ The improved usability features of CamAPS FX may therefore help to address age-related health disparities experienced by adolescents who have type 1 diabetes. Adolescent participants described feeling less shackled and burdened by diabetes by virtue of using a system with components which were easy to carry and/or wear. Additional quality-of-life benefits resulted from no longer needing to undertake finger-prick checks and/or respond to requests from parents to provide information about glucose levels. Participants also described benefiting from using a control algorithm that resided on a smartphone app, as this enabled them to check glucose and administer insulin effortlessly and in ways that did not draw attention to them having diabetes; the ability to customize and deactivate alarms was also praised in this regard. Adolescents further highlighted how self-management practices and sense of control over their condition had been aided by being able to easily review glucose data and adjust closed-loop insulin delivery using the “boost” and “ease-off” functions. While the new system addresses many of the accessibility and usability issues reported by users of previous iterations of the technology, it could potentially result in adolescents forgetting about diabetes and neglecting to undertake some key tasks.

Studies have shown how family conflicts can result when adolescents neglect to perform tasks such as checking blood glucose levels, which can act as barriers to achieving optimal glycemic control.^37,38^ As our findings illustrate, adolescents using this closed-loop system may experience lessened family conflict because of no longer needing to do finger-pricks and parents being able to monitor their glucose data remotely; the latter benefit has also been reported by CGM users and their caregivers.^38,39^ Research involving adolescents using MDI and insulin pump regimens has also highlighted how self-management tasks, such as regular finger-prick checks and administering insulin, can be neglected due to wanting to avoid unwanted attention from peers and resulting stigma.^14,40-44^ As well as being able to perform these tasks discreetly by virtue of the app being on a smartphone, participants described having checked their data more frequently because they did so opportunistically when using their smartphone for other reasons (eg, sending texts).

As others have shown, adolescents who do not receive appropriate support from teachers can hide or neglect diabetes tasks at school.^45,46^ While participants in our study described benefitting from using a medical app on an unlocked smartphone, some also highlighted challenges to accessing and using this aspect of the technology in school settings where use of smartphones is not normally permitted. Similar issues might also arise for other groups, such as adults working in customer-facing roles, where use of smartphones may not be permitted or considered appropriate. It has been suggested that users be given the choice of using a phone-based or pump-based control system according to their needs.^47^ To maximize accessibility, developers of future systems that use an app-based controller could consider providing users with the option to switch to a pump-based control system when in school and other settings where use of phones is discouraged. In addition, current guidelines^48,49^ should be updated to ensure school staff are provided with training to facilitate better understanding and provision of support to young people using closed-loop systems and diabetes smartphone apps.

In earlier studies, adolescents have reported seeking ways to communicate with and customize closed-loop systems, including being able to announce plans for physical activity or atypical days,^13,31^ or reduce “alarm fatigue.”^13,31^ As our findings illustrate, participants noted particular benefits to being able to collaborate with the closed-loop system. This included using the “boost” function to better address hyperglycemia at mealtimes or if they were unwell, and the “ease-off” function, which helped lessen anxieties about developing hypoglycemia when doing physical activity. Similar to findings from previous studies,^13,30^ we have illustrated how the reduced frequency of, and ability to personalize, alarms resulted in participants experiencing better sleep and improved quality of life due to less disruption at school, when in bed or in public settings. Importantly, our findings suggest that users may be more likely to respond to alarms when they can customize glucose thresholds and choose which ones to activate.

A key study strength is that it involved adolescents with ≥6 month’s experience using a closed-loop system in “real-life” settings, which provided a greater level of insight than earlier studies of shorter durations.^26,27,29,30,50,51^ Although our sampling took account of adolescents’ parents’ occupations, a potential limitation is our lack of representation of individuals from minority ethnic backgrounds, which reflected low representation of these groups in the wider trial. This might limit the generalizability of some of our findings. It should also be noted that our study, like others,^50^ included participants who described how the closed-loop system lessened the burdens of self-management to the extent that they could sometimes forget they had diabetes, resulting in them neglecting to perform some key diabetes tasks. Consequently, future studies could use longer periods of follow-up to establish whether these issues manifest and persist when individuals use closed-loop systems over time. It should also be noted that our sample comprised participants who had used a closed-loop since diagnosis. Hence, future research could consider individuals who have transitioned from MDI or insulin pump regimens, who, in light of these previous experiences, may perceive other or additional benefits to using closed-loop technology. Further research could also explore the views and experiences of health professionals involved in delivering care and providing support to users of the CamAPS FX system.

## Conclusions

In conclusion, our study has demonstrated how a closed-loop platform with small components and a control interface hosted on a smartphone app can help to optimize glycemic benefits and address accessibility and usability issues experienced by adolescent users of earlier iterations of the technology. Our findings also identify some challenges to using a medical app on a device which doubles as a personal smartphone. To maximize accessibility and usability among adolescents who use closed-loop systems, and consequently to help address the health-related disparities experienced by this age group, we recommend that future iterations incorporate or retain the features described above.
